# Mushroom‐shaped structures formed in *Acinetobacter baumannii* biofilms grown in a roller bioreactor are associated with quorum sensing–dependent Csu‐pilus assembly

**DOI:** 10.1111/1462-2920.15985

**Published:** 2022-03-30

**Authors:** Manuel Romero, Celia Mayer, Stephan Heeb, Krittanont Wattanavaekin, Miguel Cámara, Ana Otero, Paul Williams

**Affiliations:** ^1^ National Biofilms Innovation Centre, Biodiscovery Institute and School of Life Sciences University of Nottingham Nottingham UK; ^2^ Instituto de Investigacion Sanitaria de Santiago de Compostela (IDIS) Santiago de Compostela Spain; ^3^ Sakaeo Crown Prince Hospital Department of Internal Medicine Sa Kaeo Thailand; ^4^ Departamento de Microbioloxía e Parasitoloxía, Facultade de Bioloxía, Edificio CIBUS Universidade de Santiago de Compostela Santiago de Compostela Spain

## Abstract

There is currently a need to develop simple biofilm models that facilitate investigation of the architecture/biology of mature bacterial biofilms in a consistent/standardized manner given their environmental and clinical importance and the need for new anti‐biofilm interventions. This study introduces a novel biofilm culture system termed the rolling biofilm bioreactor (RBB). This easily operated system allows adherent microbial cells to be repeatedly exposed to air/solid/liquid interfaces optimizing biofilm growth. The RBB was exploited to investigate biofilm formation in *Acinetobacter baumannii*. High levels of *A*. *baumannii* biofilm biomass reproducibly accumulate in the RBB and, importantly, undergo a maturation step to form large mushroom‐shaped structures that had not been observed in other models. Based on image analysis of biofilm development and genetic manipulation, we show how *N*‐acylhomoserine lactone‐dependent quorum sensing (QS) impacts on biofilm differentiation, composition and antibiotic tolerance. Our results indicate that extracellular DNA (eDNA) is a key matrix component in mature *Acinetobacter* biofilms as the mushroom‐like structures consist of dense cellular masses encased in an eDNA mesh. Moreover, this study reveals the contribution of QS to *A*. *baumannii* biofilm differentiation through Csu pilus assembly regulation. Understanding the mechanisms of structural development of mature biofilms helps to identify new biofilm eradication and removal strategies.

## Introduction

*Acinetobacter baumannii* has emerged globally as one of the most troublesome multi‐antibiotic resistant hospital‐acquired pathogens due to its ability to settle and survive on surfaces after contamination via colonized patients (Jones *et al*., 2006; Dijkshoorn *et al*., 2007; Adams *et al*., 2011). Although the risk of healthy individuals developing *Acinetobacter* infection is low, clinical conditions such as open wounds, diabetes, compromised immunity, chronic lung disease, the use of ventilators and catheters as well as extended stays in hospital all significantly increase the risk of *A*. *baumannii* infection (Garnacho‐Montero and Timsit, 2019). One of the major characteristics of *A*. *baumannii* is its multi‐resistance to diverse antibiotics (Moubareck and Haummoudi Halat, 2020) including carbapenems and third‐generation cephalosporins (López *et al*., 2017). To aggravate the situation, *A*. *baumannii* forms biofilms, self‐sustaining communities of bacteria that usually form on surfaces and are highly tolerant to desiccation, nutrient starvation and antimicrobial treatment (Gaddy and Actis, 2009). Overproduction of extracellular polymeric substances (EPS) in the biofilm entrap and limit diffusion of certain antibiotics (Daddi Oubekka *et al*., 2012) while local microenvironmental conditions induce physiological changes that lead to antimicrobial tolerance and the emergence of persister cells. Moreover, potential virulence genes as well as those involved in antibiotic resistance are highly expressed in *A*. *baumannii* biofilms (Martí *et al*., 2011; He *et al*., 2015).

A complex regulatory network that integrates intra‐ and extracellular signalling systems control the expression of genes involved in biofilm formation in *A*. *baumannii* (Niu *et al*., 2008; Gaddy and Actis, 2009; Eze *et al*., 2018; López‐Martín *et al*., 2021). Among these, quorum sensing (QS), a regulatory mechanism governing the expression of diverse genes in a population density‐dependent manner, plays a key role (Niu *et al*., 2008; Mayer *et al*., 2020a; López‐Martín *et al*., 2021). *Acinetobacter baumannii* possesses an RXI‐type QS network involving AbaI, required for the synthesis of the QS signal molecule *N*‐(3‐hydroxydodecanoyl)‐l‐homoserine lactone (OHC12‐HSL), its cognate receptor, AbaR and a negative regulator, AbaM (Mayer *et al*., 2018, 2020a; López‐Martín *et al*., 2021).

In previous studies, we have provided evidence that a functional QS system is involved in surface‐associated motility and biofilm formation in *A*. *baumannii* (Mayer *et al*., 2018, 2020b; López‐Martín *et al*., 2021). Moreover, a link between QS regulation and Csu pili assembly, a type I pilus appendage important for biofilm formation in *A*. *baumannii* (Moon *et al*., 2017; Pakharukova *et al*., 2018), has been established (Luo *et al*., 2015; Mayer *et al*., 2020b; López‐Martín *et al*., 2021). Although various biofilm models have enabled quantification of differences in biofilm formation (Mayer *et al*., 2018, 2020b), multi‐layered, three‐dimensional architecture in *A*. *baumannii* biofilm communities have rarely been observed in static multiwell plates assays or on tubes. These assays rely mainly upon biomass quantification using crystal violet staining and do not allow analysis of biofilm community, spatial organization composition or architecture. Here we describe the design and application of a novel biofilm culture setup incorporating high levels of aeration, flow and access to the solid/air/liquid interfaces required to stimulate the growth of robust, mature and differentiated *A*. *baumannii* biofilms. Using this system, we demonstrate the role of QS and Csu pili on biofilm composition, architecture and tolerance to antibiotics.

## Results

### Optimisation of mature biofilm formation by *A*. *baumannii* ATCC17978 in the RBB

To identify parameters required for the transition of *A*. *baumannii* ATCC17978 from surface monolayers of scattered single cells to mature biofilms and to explore the contribution of QS, we initially employed Bioflux microfluidic chambers (Fluxion Biosciences). *Acinetobacter baumannii* biofilms were cultured in a low salt medium (YLB) (LS‐LB, Mayer *et al*., 2018) to stimulate *N*‐acylhomoserine lactone (AHL) production. Despite changes introduced with respect to inoculum size, flow/shear stress, no differentiated biofilms developed in this system. Single cells and small discrete micro‐colonies (~5–40 μm) were observed attached to the walls of the micro‐channels. Greater numbers and larger micro‐colonies were present at the air–liquid interface in the microfluidic chambers (probably as a consequence of air bubbles) (Supplementary Fig. 1). This finding is in agreement with Mayer *et al*. (2018, 2020b) who observed significantly greater biofilm formation by ATCC17978 at the air–liquid interphase under static conditions in a modified Amsterdam Active Attachment biofilm cultivation model (Muras *et al*., 2020), compared with biofilms that formed on the bottom of microtiter plate wells. Together these observations suggested that robust aeration may be the key to mature biofilm formation by *Acinetobacter*.

**Fig. 1:**
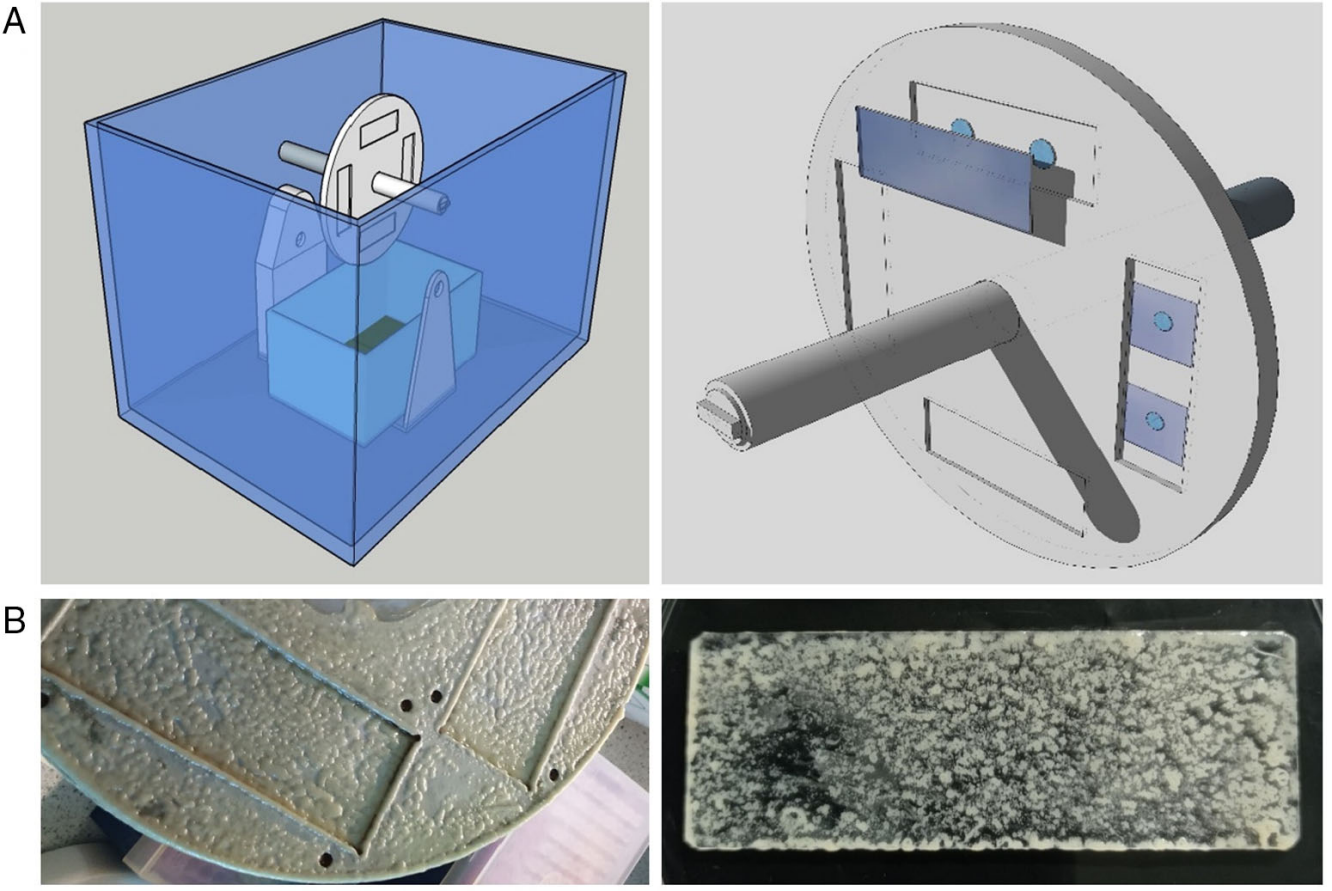
A. Schematic of the RBB setup. A detail of the spinning disk with attached glass slide/coverslips is shown in the right‐hand panel. B. Images showing mature 4‐day *Acinetobacter* biofilms growing on the RBB disk and glass microscope slide (75 mm × 25 mm).

To explore this premise, a new biofilm bioreactor system was designed and constructed. It consisted of a rotator that vertically spins a wheel to which abiotic surface substrata can be attached enabling periodic dipping into a culture allowing adherent cells to be repeatedly aerated so optimizing biofilm growth. We termed this system the Rolling Biofilm Bioreactor (RBB) (Fig. 1A). Under conditions that are best for strictly aerobic bacterial species, ATCC17978 produced robust macroscopic 3D biofilms even on glass (Fig. 1B), a material previously reported to be the least favourable substratum for biofilm formation by *A*. *baumannii* (Greene *et al*., 2016).

### QS influences *A*. *baumannii* biofilm biomass and architecture

**Fig. 2:**
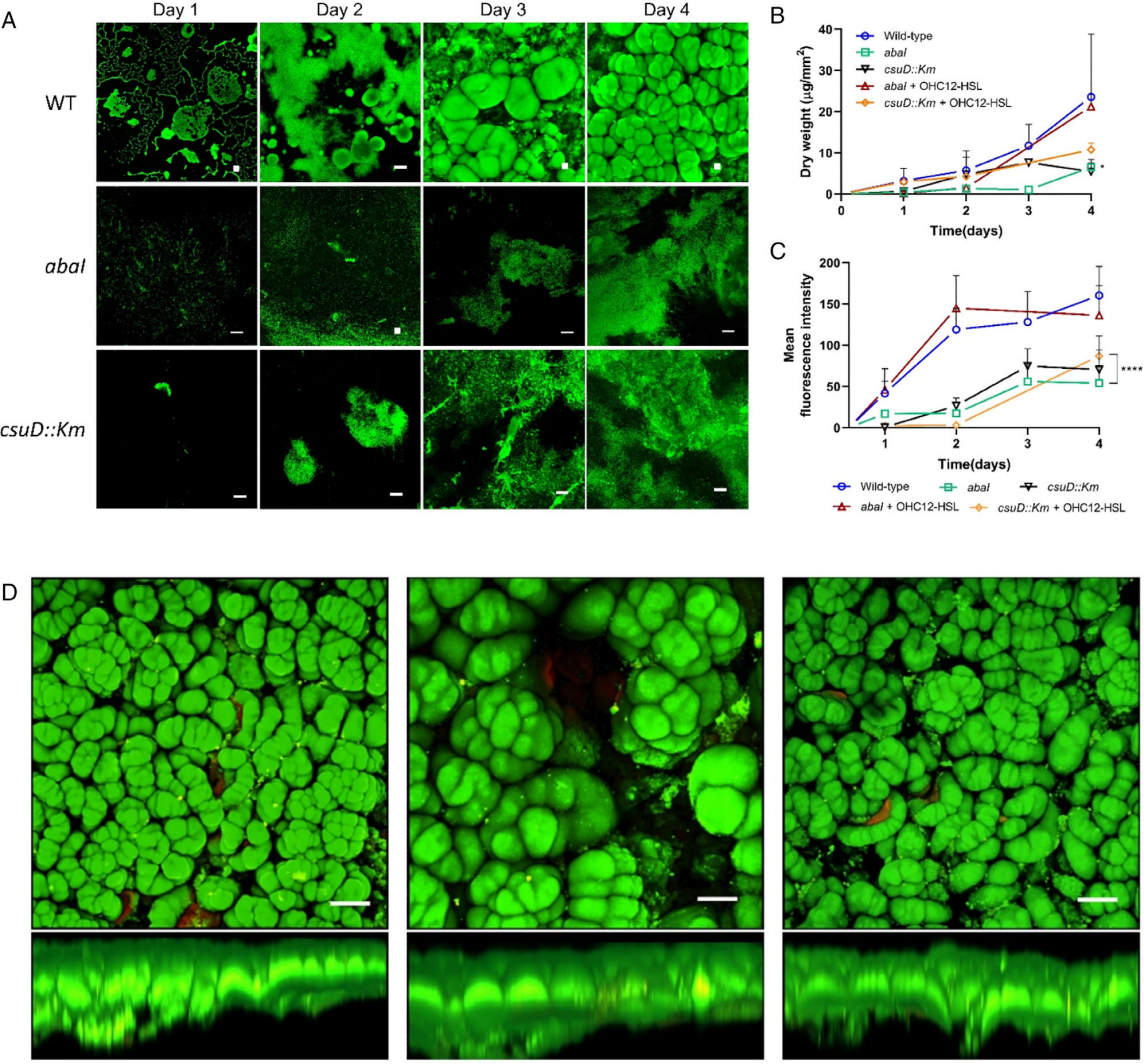
A. Representative CLSM images comparing biofilm development by *A*. *baumannii* wild‐type, *abaI* mutant and *csuD* pilus mutant after 1–4 days incubation in the RBB and stained with Syto9. Scale bar: 50 μm. B. RBB biofilm dry weight measurement and (C) quantification of mean fluorescence intensity in biofilm cultures (±10 μM OHC12‐HSL supplementation). Data shown are mean ± SD. Statistical significance was determined with multiple *t* tests using the Holm–Sidak method (*p < 0.05; **p < 0.01; ***p < 0.001; ****p < 0.0001). D. Representative 3D CLSM images of ATCC17978 wild‐type strain biofilms obtained after 4 days incubation in the RBB and stained with a live/dead bacterial viability kit. Scale bar: 100 μm.

Using the RBB, the biofilm growth of an AHL‐deficient *A*. *baumannii abaI* mutant and its isogenic parent was quantified daily over a 4‐day period. Biofilm dry weight measurements and confocal laser scanning microscope (CLSM) imaging revealed that the early micro‐colonies and cell aggregates of the wild type ATCC17978 transitioned to multi‐layered biofilms from day 2 (Fig. 2A). Notably, after 3 days of incubation, macroscopic ‘mushroom‐shaped’ structures emerged. To our knowledge, these almost spherical three‐dimensional structures with a characteristic central depression have not previously been reported in *Acinetobacter*. The cell clusters were approximately 100–300 μm in diameter and 100–200 μm thick. The macro‐colonies continued to grow and became confluent forming a complex, thick (>300 μm) biofilm mass by day 4 in the wild‐type strain (Fig. 2A–D). Most of the substratum was covered with cell clusters and open areas with reduced biofilm thickness (~10–20 μm) were observed between the macro‐colonies. Similar to mature biofilms produced by undefined microbial consortia (de Beer *et al*., 1994), microscopic observations revealed voids between the larger cell mushroom‐like structures and the substratum indicating that the clusters were not closely attached to the glass substrate giving the *A*. *baumannii* community a ‘spongy’ appearance (Fig. 2D).

Compared with the wild‐type, and as previously reported by crystal violet staining biofilms produced on glass coverslips (Mayer *et al*., 2020a), the *A*. *baumannii abaI* mutant formed substantially less biofilm under the highly aerated flow conditions within the RBB, with 3.6‐times less dry biofilm weight measured after 4 days of sessile growth on glass compared with the isogenic parental strain (Fig. 2A–C). Moreover, other changes in the biofilm architecture were also observed. The *abaI* mutant biofilms consisted mainly of undifferentiated cellular aggregates with a few small and discrete macro‐colonies (Fig. 2A). The addition of exogenous OHC12‐HSL to the *abaI* mutant in RBB cultures resulted in recovery of the biofilm biomass yields (Fig. 2B and C), although smaller mushroom‐shaped structures were observed compared with those obtained for the wild‐type strain (Supplementary Figs 2 and 3). We attributed this to the low solubility of OHC12‐HSL when supplemented to fresh medium at 10 μM since this hydrophobic QS signal molecule was observed to precipitate out of solution after inoculation at this concentration. Therefore the more soluble compound *N*‐(3‐hydroxydecanoyl)‐l‐homoserine lactone (OHC10‐HSL), a second AHL produced by ATCC17978 although detected in much minor amounts (Mayer *et al*., 2018), was also tested. When supplied at 1 μM, OHC10‐HSL chemically complemented biofilm macro‐colony formation from day 2 post culture of the *abaI* mutant (Supplementary Fig. 2). However, OHC12‐HSL provided at 1 μM was unable to fully restore mushroom macro‐colony formation (Supplementary Fig. 3). These data strongly support a role for QS in *A*. *baumannii* biofilm maturation.

### Csu pili and biofilm maturation in *A*. *baumannii*

Previous studies have shown that inhibition of the chaperone/usher pili‐associated *csu* operon, encoding a single six‐gene operon (*csuA/B*, *csuA*, *csuB*, *csuC*, *csuD* and *csuE*) responsible for the assembly and extension of type I pilus in *A*. *baumannii*, results in a significant reduction in biofilm formation (Moon *et al*., 2017; Pakharukova *et al*., 2018). Furthermore, links between *csu* expression and QS regulation have been established in this pathogen (Luo *et al*., 2015; Mayer *et al*., 2020b; López‐Martín *et al*., 2021). To assess the importance of Csu pili for biofilm differentiation and provide mechanistic understanding of how QS regulation affects biofilm architecture and biomass, the Csu pili deficient *A*. *baumannii* ATCC17978 mutant *csuD*::*kan* was cultured in the RBB. Previous analyses have provided evidence that CsuD together with CsuC function as a chaperone‐usher secretion machinery that assembles the four type I pilus subunits, namely, CsuA/B, CsuA, CsuB and CsuE (Tomaras *et al*., 2003, 2008).

In common with the *abaI* mutant, the *csuD* mutant failed to form the differentiated biofilm architecture under highly aerated flow conditions characteristic of the wild‐type (Fig. 2A–C). This suggested that the absence of mature mushroom‐like structures in the *abaI* QS mutant is likely to be associated with reduced expression of the *csu* operon and would be in agreement with previous reports showing an increase in *csuD* expression under the same conditions that induce *abaI* expression and AHL synthesis (Mayer *et al*., 2018) and a reduction in cell surface pili in an *A*. *baumannii* ATCC17978 *abaI* mutant (Mayer *et al*., 2020b). In contrast to the *abaI* mutant, addition of exogenous OHC12‐HSL to the *csuD*::*kan* mutant cultures did not result in the recovery of biofilm biomass (Fig. 2B and C; Supplementary Fig. 2). Since QS regulates *csu* expression, these data provide further support that Csu pili are essential for biofilm maturation in *A*. *baumannii* ATCC17978 under these growth conditions.

To further explore the link between QS/Csu expression and biofilm maturation, genetic complementation of mushroom‐like structures formation in the *abaI* mutant was attempted by introducing the plasmid pBAV1K‐T5‐*csu* (P*csu*) harbouring a constitutively expressed *csu* operon. Interestingly, higher biofilm biomass and mushroom‐shaped structures were produced by the *abaI* mutant when expressing the *csu* operon independent of QS control (Supplementary Fig. 4A and B). Likewise, the expression of the same operon in *E*. *coli* strain DH5α increased the biomass of the parental strain with macrocolonies starting to form in the biofilm from day 2 in the RBB system (Supplementary Fig. 4).

### *Acinetobacter baumannii* ATCC17978 biofilm composition

**Fig. 3:**
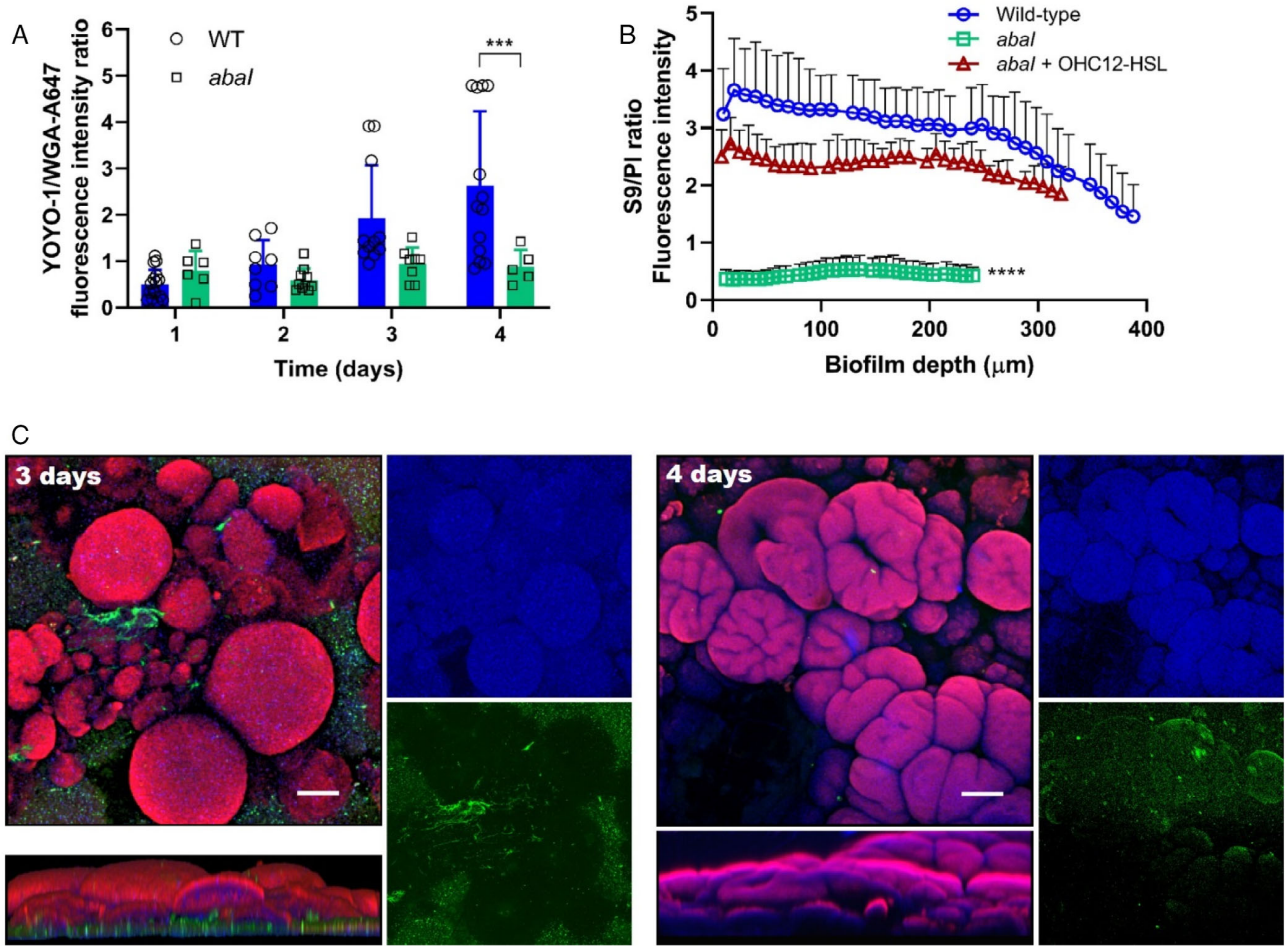
A. Quantification of mean fluorescence intensity ratios for YOYO1 iodide (eDNA) and Wheat Germ Agglutinin Alexa Fluor 647 conjugate (WGA‐A647; PNAG exopolysaccharide) stained biofilms of *A*. *baumannii* wild‐type and *abaI* mutant strains after 1–4 days incubation in the RBB. B. Profiles of live/dead ratios of *A*. *baumannii* wild‐type and *abaI* mutant (±10 μM OHC12‐HSL) biofilms grown in the RBB for 4 days. Data shown are mean ± SD. Statistical significance was determined with multiple *t*‐tests using the Holm–Sidak method (*p < 0.05; **p < 0.01; ***p < 0.001; ****p < 0.0001). C. CLSM images of 3‐ and 4‐day‐old biofilms stained with YOYO1 (blue), WGA‐A647 (green) and FM4‐64 (red) fluorogenic dyes to mark eDNA, PNAG polysaccharide and cell membranes respectively. Scale bar: 100 μm. Bottom panels: side volumetric 3D projections of the biofilms.

In contrast to conventional biofilm models, the RBB system provided an opportunity to determine the spatial organization and architecture of mature, differentiated *A*. *baumannii* biofilms. To this end, staining for EPS biofilm components, extracellular DNA (eDNA) and poly‐ß‐(1‐6)‐N‐acetyl‐glucosamine (PNAG), combined with CLSM analysis was employed. Our results revealed that eDNA is a key matrix component in differentiated *Acinetobacter* biofilms. eDNA ‘webs’ were detected in early‐stage biofilms (Fig. 3A and C). The mushroom‐like structures consisted of dense cellular masses [stained with FM4‐64 (red)] encased in a mesh of eDNA as shown in blue by YOYO‐1 staining (Fig. 3A and C). On the other hand, PNAG was observed as patchy green aggregates in the bottom layers of the biofilm between the mushroom‐like structures when stained with the WGA‐A647 fluorochrome. This suggested that this polymer could have a lesser function in the architecture of these macro‐colonies compared to eDNA (Fig. 3A and C). Moreover, a gradual increase in the eDNA content of the biofilm matrix compared with PNAG observed during biofilm maturation highlighted a role for eDNA in the architecture of the mature biofilm macro‐colonies (Fig. 3A). Interestingly, the eDNA to PNAG ratios remained constant in the *abaI* mutant biofilms grown for 4 days (Fig. 3A). These lacked mushroom‐like structures (Fig. 2A) further indicating a potential scaffolding role for eDNA.

Cell viability staining with SYTO9 and propidium iodide showed that the upper layers of wild‐type biofilms were composed mainly of live cells, with a smaller number of dead cells in the bottom layers (Figs 2D and 3B). Remarkably, the live/dead profiles of the QS deficient *abaI* mutant revealed a large amount of cell debris/dead cells throughout the depth of a 4‐day biofilm. However, supplementation of the *abaI* mutant with OHC12‐HSL restored viability almost to wild‐type levels throughout the 4‐day biofilm (Fig. 3B; Supplementary Fig. 3). Similarly, live/dead ratios of the *abaI* mutant could be restored to wild‐type levels in communities growing in Yersinia–Luria–Bertani (YLB) supplemented with this AHL over the 4‐day period (Supplementary Fig. 3B).

### Mature biofilms of *A*. *baumannii* ATCC17978 display greater tolerance to kanamycin

**Fig. 4:**
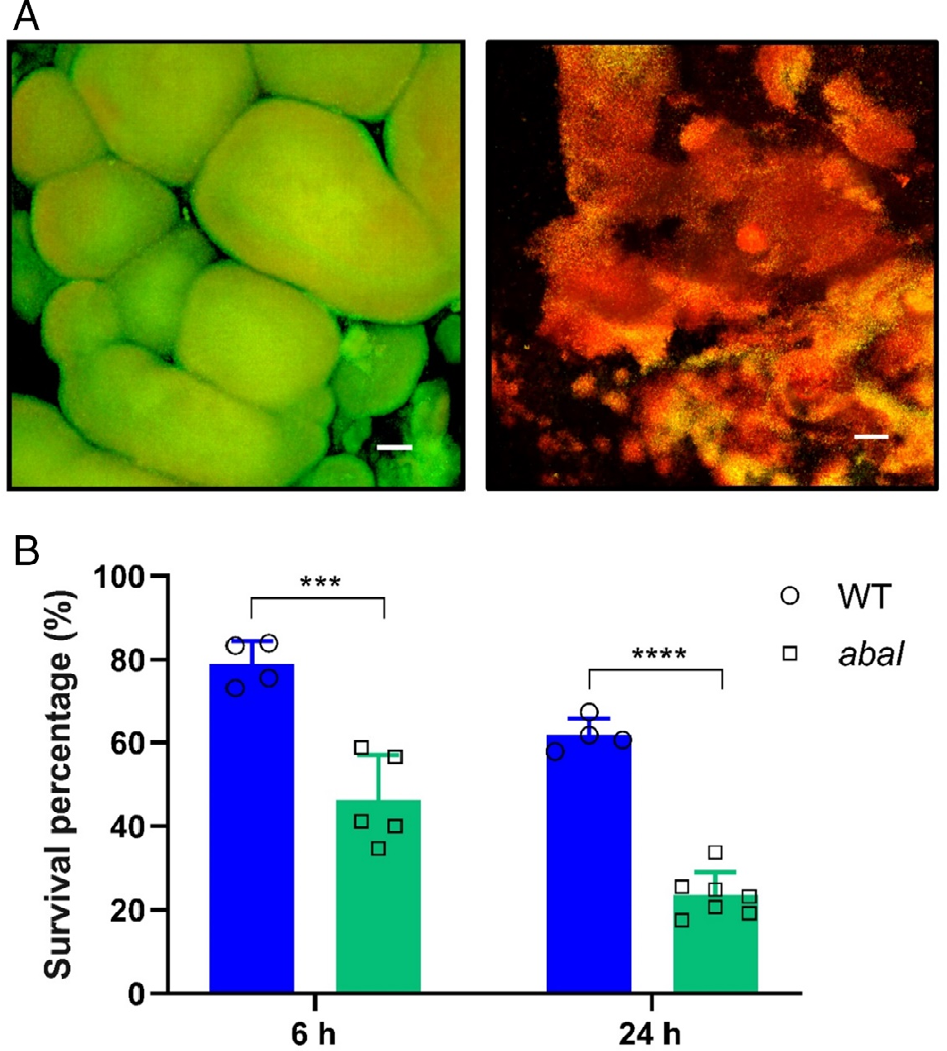
A. CLSM images of 4‐day‐old biofilms of wild‐type *A*. *baumannii* (left) and *abaI* mutant (right) strains after 24 h treatment with 1.5 mg ml^−1^ kanamycin and stained with live/dead bacterial viability kit. Scale bar: 50 μm. B. 4 day‐old biofilm viability after 6 and 24 h kanamycin exposure respectively quantified as live/dead mean fluorescent ratios of wild‐type and *abaI* strains and normalized to untreated controls. Data shown are mean ± SD. Statistical significance was determined with multiple *t*‐tests using the Holm–Sidak method (*p < 0.05; **p < 0.01; ***p < 0.001; ****p < 0.0001).

To determine whether mature biofilms of the *A*. *baumannii* wild‐type showed a higher tolerance to antibiotics than the *abaI* mutant, 4‐day biofilms grown in the RBB system were exposed to 300× MIC, i.e. at 1.5 mg ml^−1^ of kanamycin for 6 and 24 h respectively. Live and dead cell staining of the antibiotic‐treated biofilms showed a clear reduction in antibiotic tolerance by the *abaI* biofilms compared with the parental strain (Fig. 4A and B) suggesting that the inability of the mutant to produce mature, organized biofilms renders it more susceptible to antimicrobials.

## Discussion

Difficulties in obtaining mature *A*. *baumannii* biofilms in static biofilm models have resulted in studies exploring the role of AHL‐mediated QS systems in biofilm development focusing mainly on the early stages (Niu *et al*., 2008; Kang and Park, 2010; Anbazhagan *et al*., 2012). However, developing differentiated *Acinetobacter* biofilms is important as biofilm maturity impacts on antibiotic tolerance (Tré‐Hardy *et al*., 2009) and the molecular composition and architecture of the biofilm community. Moreover, a detailed understanding of the developmental process from single scattered cells attached to a substratum to the formation of an organized multicellular biofilm is essential for developing novel biofilm management strategies. Here we introduce a new and simple to set up biofilm culturing system, the RBB, which facilitates the reproducible formation of thick mature and complex *A*. *baumannii* biofilms. These are useful for investigating the morphological, organizational and functional changes taking place during different stages of the biofilm maturation including those that occur in response to antimicrobial treatment. Indeed, our results show that biofilms of *A*. *baumannii* grown in the RBB setup can be used to define the role(s) of QS in the formation and maturation of biofilm communities (Niu *et al*., 2008; Kang and Park, 2010; Mayer *et al*., 2020b). Using the RBB model we show that QS controls morphological changes in the late phases of biofilm development. Moreover, our research confirms the link between QS regulation of Csu pili (Luo *et al*., 2015; Mayer *et al*., 2020b; López‐Martín *et al*., 2021), providing further mechanistic insights into how cell‐to‐cell signalling may affect the architecture of mature *A*. *baumannii* biofilms. An important advantage of the RBB system is the possibility of growing biofilms in highly aerated conditions that are optimal for aerobic microbial species. Moreover, this biofilm culture method is more straightforward and requires less equipment and personnel training to set up compared with other flow systems. A weakness is the volume of medium needed to allow mature biofilm development in the RBB setup (~1.2 L ^−1^ day^−1^); however, this disadvantage could be overcome by RBB designs employing wheels with more reduced diameters than the one used in this study and, therefore, smaller volume tanks could be used.

Previous bacterial biofilm development studies have shown the involvement of adhesive organelles in the maturation of these communities, particularly cell surface‐associated fimbriae assembled through conserved chaperone/usher pathways. Giraud *et al*. (2011) identified a locus in *Pseudomonas aeruginosa*, named *cupE* and with homology to the *csu* operon of *A*. *baumannii*, which was found responsible not only for the biosynthesis of pili involved in adhesion to abiotic substrata but also in cell clustering and the formation of mushroom‐shaped structures during biofilm growth. This feature appears to be shared by other chaperone/usher fimbrial paralogues in *P*. *aeruginosa*, such as the pilus assembly proteins CupB and C that also contribute to cohesive cell–cell bonding and microcolony formation in the biofilm differentiation process (Ruer *et al*., 2007). Their incomplete assembly or absence has a major impact on 3D mushroom microcolony formation. Our results support this paradigm in *A*. *baumannii* ATCC17978 since *csuD* mutants exhibited impaired Csu pilus biogenesis (Moon *et al*., 2017) while here we found that the *csuD* mutant was incapable of biofilm differentiation and produced biofilms of only monolayers of scattered cells, whereas thick cell clusters with spherical shapes were observed in the wild‐type strain.

Consistent with earlier reports, AHL supplementation of *A*. *baumannii abaI* mutant resulted in the recovery of biofilm yields to levels similar to the wild‐type strain (Luo *et al*., 2015). Strikingly, our microscopic observations of mature *Acinetobacter* biofilms showed that OHC12‐HSL only partially restored the formation of mushroom‐shaped cell clusters while supplementation with the second AHL OHC10‐HSL produced by ATCC17978 (Mayer *et al*., 2018) induced fully development of these complex structures. This could be related to the different solubilities observed for both signals, differential functionalities for the two structurally distinct AHLs or that fine‐tuning of pilus production by QS may be needed for macrocolony formation. This is supported by the fact that irregular and more discrete macrocolonies were observed in the *abaI* mutant when expressing the *csu* operon from a constitutive promoter. Remarkably, mushroom‐like structures could be achieved in *E*. *coli* expressing Csu pili and grown in the RBB, further supporting the involvement of these appendages in macrocolony formation under hydrodynamic conditions.

Comparative compositional analysis of 4‐day‐old biofilms of the *abaI* mutant and parental strain grown in the RBB suggests that eDNA release is an important factor for biofilm transition from monolayers of scattered cells to mushroom‐shaped multicellular structures. Lysis of cell subpopulations in bacterial biofilms is known to release eDNA to tie cells in clusters and enable remodelling of the extracellular matrix by promoting attractive acid–base interactions (Ibáñez de Aldecoa *et al*., 2017). eDNA has also been associated with stabilizing *A*. *baumannii* biofilm communities (Tetz *et al*., 2009). Notably, QS regulatory circuits and coordinated eDNA release in biofilms are frequently linked in bacteria (Spoering and Gilmore, 2006; Yang and Lan, 2016). Known QS‐dependent mechanisms of eDNA release include prophage activation and biosynthesis of phenazines that induce cell lysis and release of large amounts of eDNA (Allesen‐Holm *et al*., 2006; Das and Manefield, 2012, 2013). Further studies are required to determine whether there is a direct link between QS regulation and eDNA release in biofilms of *A*. *baumannii*.

QS has also been linked to antibiotic tolerance in bacterial biofilms (Jakobsen *et al*., 2017). Furthermore, enzymatic quenching of QS has been shown to increase the susceptibility of *A*. *baumannii* monospecies biofilms to antibiotics (Zhang *et al*., 2017). Here we show that mature 4‐day old biofilms of *A*. *baumannii* ATCC17978 display notable resistance to the aminoglycoside kanamycin compared to the non‐differentiated biofilm monolayers formed by a QS deficient mutant of the same strain. This could be linked to the fact that sessile cells of *A*. *baumannii* in late stages of biofilm development are tethered to an eDNA‐rich extracellular matrix since this nucleic acid has been documented to contribute to increased resistance to aminoglycosides antibiotics, including kanamycin (Wilton *et al*., 2015). One possibility is that the activities of antimicrobial agents are quenched on sequestration by eDNA functioning as a physical barrier preventing their access to cellular targets (Chiang *et al*., 2013). Also, eDNA accumulation in local biofilm microenvironments can reduce the local pH, which constitutes a signal for the activation of pathways that lead to aminoglycoside resistance through cell envelope modifications (Wilton *et al*., 2015). Similarly, the induction of divalent cation limitation mediated by eDNA release helps protect microbial cells from positively charged antimicrobials (Johnson *et al*., 2012). Confirming the importance of eDNA as scaffold for the architecture of biofilm in this species, several reports have highlighted the potential of DNAses for increasing the susceptibility of biofilms to biocides and to reduce biofilm formation and even destabilize preformed *A*. *baumannii* communities (Tetz *et al*., 2009; Sahu *et al*., 2012; Mayer *et al*., 2020b).

The unique characteristics of the RBB system allowed us to obtain high yields of *A*. *baumannii* biomass in the form of mature biofilms in a reproducible manner and permitted the study of fundamental aspects of biofilm development by this pathogen. Based on image analysis of biofilm development by *A*. *baumannii* ATCC17978, our results revealed that the wild‐type and isogenic *abaI* mutant both attach to surfaces in the RBB system; however, the QS deficient strain cannot progress to form a mature, differentiated biofilm. These results demonstrate a central role for AHL‐based QS in the regulation of biofilm development in *A*. *baumannii* and further support the validity of efforts directed towards the disruption of QS as a promising approach to prevent and manage *A*. *baumannii* colonization and survival on surfaces, especially in combination with antibacterial agents.

## Experimental procedures

### Bacterial strains and culture conditions

*Acinetobacter baumannii* ATCC17978 wild‐type strain, *abaI* deletion mutant (Mayer *et al*., 2020b) and *csuD*::*kan* insertion mutant (Moon *et al*., 2017) were routinely grown at 37°C in lysogeny broth (LB) or LB agar supplemented with kanamycin 25 μg ml^−1^ as required. YLB broth (1% tryptone, 0.5% yeast extract and 0.5% NaCl) was used as the growth medium for biofilm assays. The *A*. *baumannii* cognate AHL signal molecules were synthesized in‐house as described before (Ortori *et al*., 2007) and used at concentrations of 1 and 10 μM for OHC12‐HSL and 1 μM for OHC10‐HSL.

### Construction of a plasmid expressing the *csu* operon

Primers csuOFw (5′AACCATGGAGATTAGCCATATTTTATTTGTCGAG3′) and csuORv (5′TTCTCGAGTTAAAGATAAAAGCCCATGAACTGAG3′), which introduce NcoI and XhoI sites respectively, were used to amplify by PCR and clone the full *csu* operon (6 kbp) from *A*. *baumannii* ATCC17978 into the plasmid pBAV1K‐T5‐*gfp* (Kan^R^, Bryksin and Matsumura, 2010). The resulting plasmid pBAV1K‐T5‐*csu* (P*csu*) was propagated in *E*. *coli* DH5α and subsequently introduced in the *abaI* mutant of ATCC17978.

### Bioflux assay

Biofilms formed under shear flow conditions were cultivated in the BioFlux™ 200 microfluidics System (Fluxion Biosciences, CA, USA) as previously described (Nait Chabane *et al*., 2014) with some modifications. Micro‐channels were primed with ATCC17978 at optical density at 600 nm (OD_600nm_) 0.01–0.05 followed by 1 h static incubation at 37°C to allow cells to attach to the channel walls. The shear flow was then started at different rates from 0.5 to 3 dyn cm^−2^ and the setup was incubated at 37°C for 16 h. Adherent cells were stained with 5 μM SYTO® 9 (Invitrogen) and imaged using a Zeiss LSM 700 CLSM (Carl Zeiss).

### Roller biofilm bioreactor

For the RBB model assembly, an incubation‐compatible lab rotator (Labnet International H5600 Revolver Rotator) was adapted. The original rotating disk was removed, and a sterile 1.5 L glass container was placed between the disk holders. Next, 1.2 L of fresh medium was inoculated with *A*. *baumannii* at OD_600nm_ 0.01 and placed in the container. A customized aluminium wheel (Fig. 1A) with sterilized glass coverslips (13 mm Ø, Menzel‐Glaser, Thermo Scientific) and/or glass slides (76 mm × 26 mm, Menzel‐Glaser, Thermo Scientific) attached was then assembled in the rotator, and all components were introduced in a sterilized closable polypropylene box. Finally, the wheel rotation (at 20 rpm) was started and the RBB incubated at 37°C for up to 4 days with medium replacement every 24 h under aseptic conditions. Biofilms attached to glass slides were air‐dried and weighed to quantify the total biofilm biomass. For CLSM analysis, biofilms on coverslips were washed by dipping in phosphate‐buffered saline (PBS) buffer and stained with SYTO 9. YOYO™‐1 iodide (Invitrogen) and Wheat Germ Agglutinin Alexa Fluor™ 647 (WGA‐A647) conjugate (Invitrogen) dyes were used to stain biofilm eDNA and poly‐ß‐(1‐6)‐N‐acetyl‐glucosamine (PNAG) respectively. Cell membranes were stained with FM™4‐64 (Invitrogen) lipophilic stain. Following staining, coverslips were rinsed with sterile water and imaged using CLSM.

### Biofilm antibiotic tolerance assay

For antibiotic tolerance assays, 4‐day biofilms grown in the RBB were exposed to 1.5 mg ml^−1^ of kanamycin (~300× their minimal inhibitory concentration – MIC) for 6 and 24 h in PBS at 37°C statically. The antibiotic MIC was defined as the concentration where no visible planktonic growth was observed or OD_600nm_ was <10% compared with the untreated control after 24 h exposure. Treated and untreated biofilms were washed with PBS, and cell viability was evaluated by fluorescent staining with the LIVE/DEAD™ BacLight™ Bacterial Viability kit (Molecular Probes, Life Technologies) following the manufacturer's instructions.

### Data analysis

Fluorescence data from CLSM images were obtained by measuring the mean fluorescence intensity (sum of the grey values of all the pixels in the images divided by the number of pixels) using the open‐source software Fiji‐ImageJ v2.1.0/1.53c (Schindelin *et al*., 2012). For z‐stack biofilm images, the Maximum Intensity Projection algorithm from Fiji‐ImageJ was used to select pixels of the highest intensity from every slice throughout the volume of the 3D image to construct a 2D image. Multiple *t*‐tests comparisons with Holm–Sidak correction were applied to determine whether mutants response differed significantly from that of the parental strain (*p* < 0.05) when compared with the variations within the replicates using GraphPad Prism 8.0 (GraphPad Software, San Diego, CA, USA).

## Authors' Contributions

M.R., A.O., P.W. and M.C. conceived the project. M.R. and C.M. designed and conducted the experiments. M.R., S.H. and K.W. contributed to designing and optimizing the rolling biofilm bioreactor system. M.R. and C.M. wrote the manuscript with input from all other authors.

## Supporting information


**Supplementary Fig. 1.** CLSM images of *A*. *baumannii* biofilms stained with Syto9 after 24 h growth under shear flow conditions in microfluidic chambers in a BioFlux 200 system in the absence (Solid–liquid interphase) or presence of air bubbles trapped within the microchannels (Solid‐air‐liquid interface). Scale bar: 20 μm.
**Supplementary Fig. 2.** Representative CLSM images comparing biofilm development by *abaI* and *csuD* pili mutants of *A*. *baumannii* supplemented with OHC12‐HSL (10 μM) or OHC10‐HSL (1 μM) after 1–4 days incubation in the RBB and stained with Syto9. Scale bar: 50 μm.
**Supplementary Fig. 3.** A) CLSM images of *A*. *baumannii* ATCC17978 wild‐type and *abaI* mutant (+/− 1 μM OHC12‐HSL) biofilms obtained after 4 days incubation in the RBB and stained with a live/dead bacterial viability kit. Scale bar: 50 μm. B) Evolution of live/dead ratios of *A*. *baumannii* wild‐type and *abaI* (+/− 1 μM OHC12‐HSL) mutant biofilms grown in the RBB or 1–4 days. Data shown are mean ± SD. Statistical significance was determined with multiple t‐tests using the Holm‐Sidak method (*p < 0.05; **p < 0.01; ***p < 0.001; ****p < 0.0001).
**Supplementary Fig. 4.** A) CLSM images of biofilm development of the *A*. *baumannii abaI* mutant strain constitutively expressing the *csu* operon (*Pcsu*) and *E*. *coli* DH5α ‐/+ csu after 2 and 3 days of incubation in the RBB and stained with Syto9. Scale bar: 50 μm. B) Quantification of mean fluorescence intensity of biofilm images from cultures of indicated strains after 3 days of incubation in the RBB and stained with Syto9. Data shown are mean ± SD. Statistical significance was determined with one‐way ANOVA (*p < 0.05; **p < 0.01; ***p < 0.001; ****p < 0.0001).
