# Supplementary material for: Mushroom‐shaped structures formed in Acinetobacter baumannii biofilms grown in a roller bioreactor are associated with quorum sensing–dependent Csu‐pilus assembly
Source: Environ Microbiol. 2022 Mar 30;24(9):4329–39. doi: 10.1111/1462-2920.15985 (PMC9790458; doi:10.1111/1462-2920.15985)
Supplement: Supplementary file 1 — Supplementary Fig. 1. CLSM images of A. baumannii biofilms stained with Syto9 after 24 h growth under shear flow conditions in microfluidic chambers in a BioFlux 200 system in the absence (Solid–liquid interphase) or presence of air bubbles trapped within the microchannels (Solid‐air‐liquid interface). Scale bar: 20 μm. Supplementary Fig. 2. Representative CLSM images comparing biofilm development by abaI and csuD pili mutants of A. baumannii supplemented with OHC12‐HSL (10 μM) or OHC10‐HSL (1 μM) after 1–4 days incubation in the RBB and stained with Syto9. Scale bar: 50 μm. Supplementary Fig. 3. A) CLSM images of A. baumannii ATCC17978 wild‐type and abaI mutant (+/− 1 μM OHC12‐HSL) biofilms obtained after 4 days incubation in the RBB and stained with a live/dead bacterial viability kit. Scale bar: 50 μm. B) Evolution of live/dead ratios of A. baumannii wild‐type and abaI (+/− 1 μM OHC12‐HSL) mutant biofilms grown in the RBB or 1–4 days. Data shown are mean ± SD. Statistical significance was determined with multiple t‐tests using the Holm‐Sidak method (*p < 0.05; **p < 0.01; ***p < 0.001; ****p < 0.0001). Supplementary Fig. 4. A) CLSM images of biofilm development of the A. baumannii abaI mutant strain constitutively expressing the csu operon (Pcsu) and E. coli DH5α ‐/+ csu after 2 and 3 days of incubation in the RBB and stained with Syto9. Scale bar: 50 μm. B) Quantification of mean fluorescence intensity of biofilm images from cultures of indicated strains after 3 days of incubation in the RBB and stained with Syto9. Data shown are mean ± SD. Statistical significance was determined with one‐way ANOVA (*p < 0.05; **p < 0.01; ***p < 0.001; ****p < 0.0001). [file EMI-24-4329-s001.docx]

**
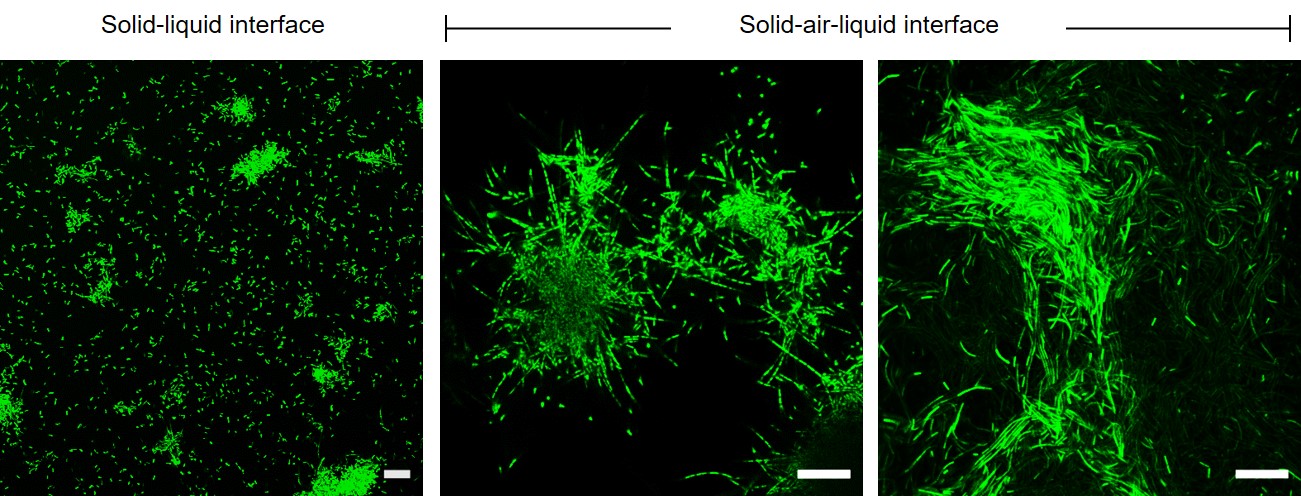
**

**Supplementary figure 1.** CLSM images of *A. baumannii* biofilms stained with Syto9 after 24h growth under shear flow conditions in microfluidic chambers in a BioFlux 200 system in the absence (Solid-liquid interphase) or presence of air bubbles trapped within the microchannels (Solid-air-liquid interface). Scale bar: 20 µm.

**
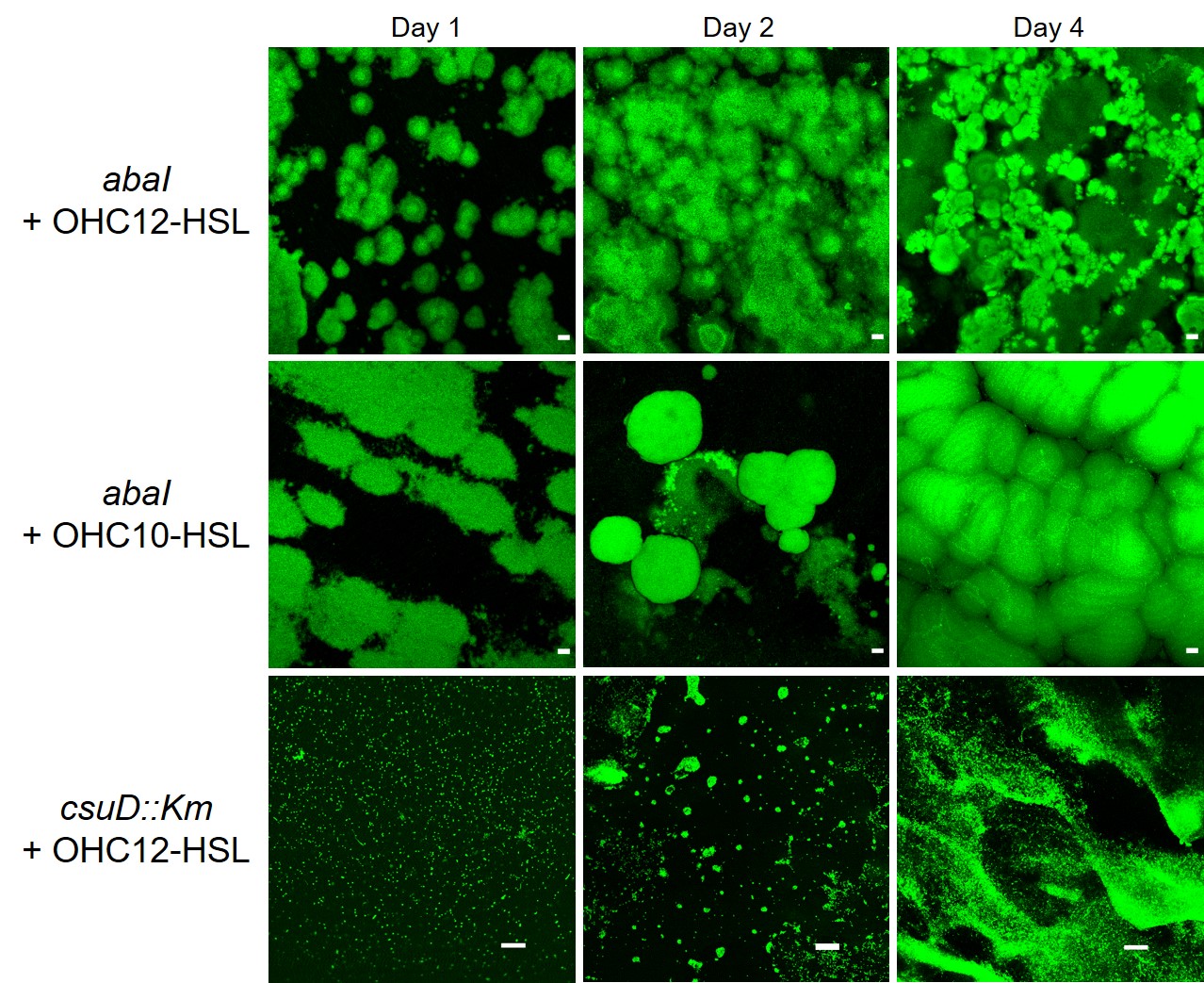
**

**Supplementary figure 2.** Representative CLSM images comparing biofilm development by *abaI* and *csuD* pili mutants of *A. baumannii* supplemented with OHC12-HSL (10 µM) or OHC10-HSL (1 µM) after 1-4 days incubation in the RBB and stained with Syto9. Scale bar: 50 µm.

**
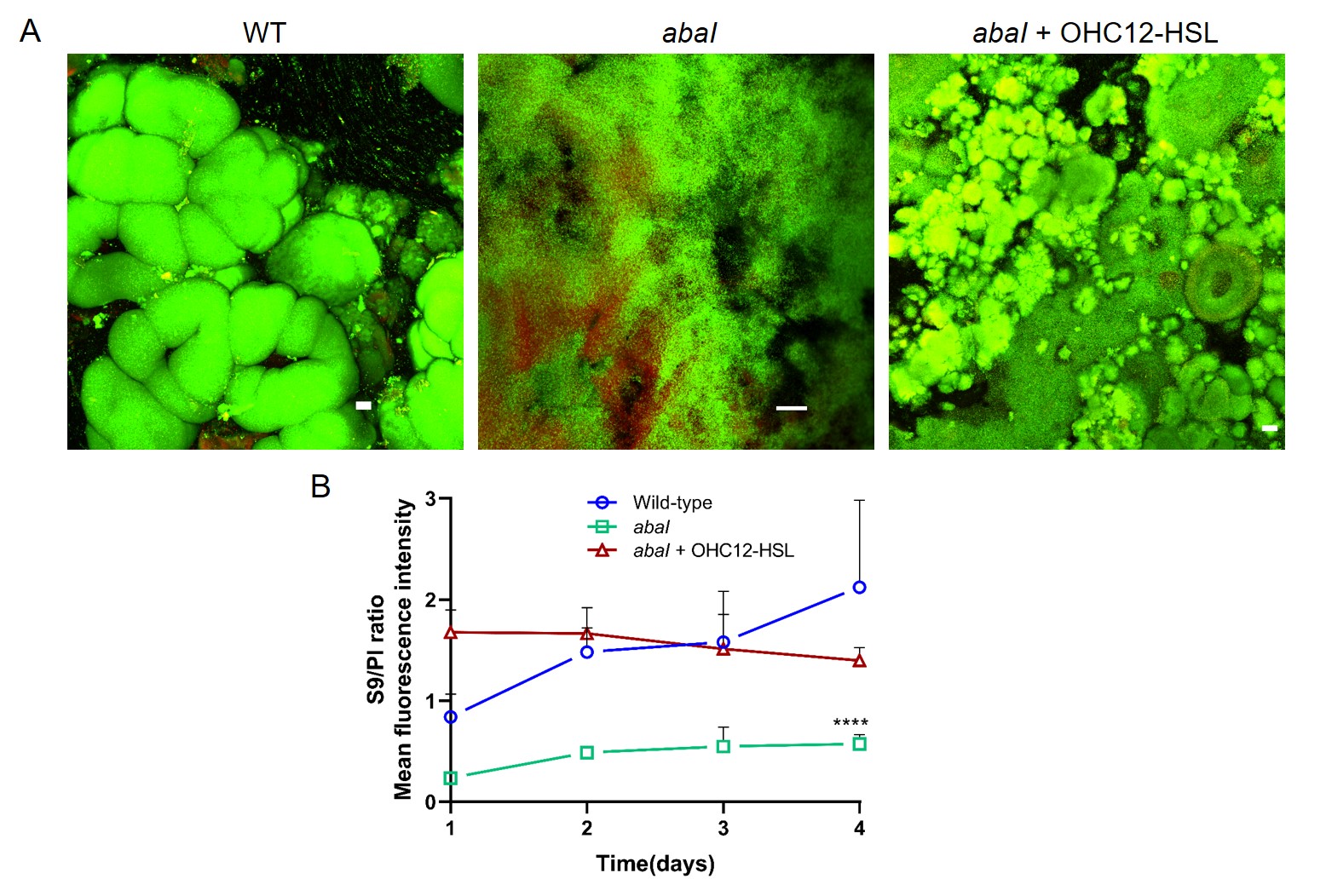
**

**Supplementary figure 3. A)** CLSM images of *A. baumannii* ATCC17978 wild-type and *abaI* mutant (+/- 1 µM OHC12-HSL) biofilms obtained after 4 days incubation in the RBB and stained with a live/dead bacterial viability kit. Scale bar: 50 µm. **B)** Evolution of live/dead ratios of *A. baumannii* wild-type and *abaI* (+/- 1 µM OHC12-HSL) mutant biofilms grown in the RBB or 1-4 days. Data shown are mean ±SD. Statistical significance was determined with multiple t-tests using the Holm-Sidak method (* p<0.05; ** p<0.01; *** p<0.001; **** p<0.0001).

**
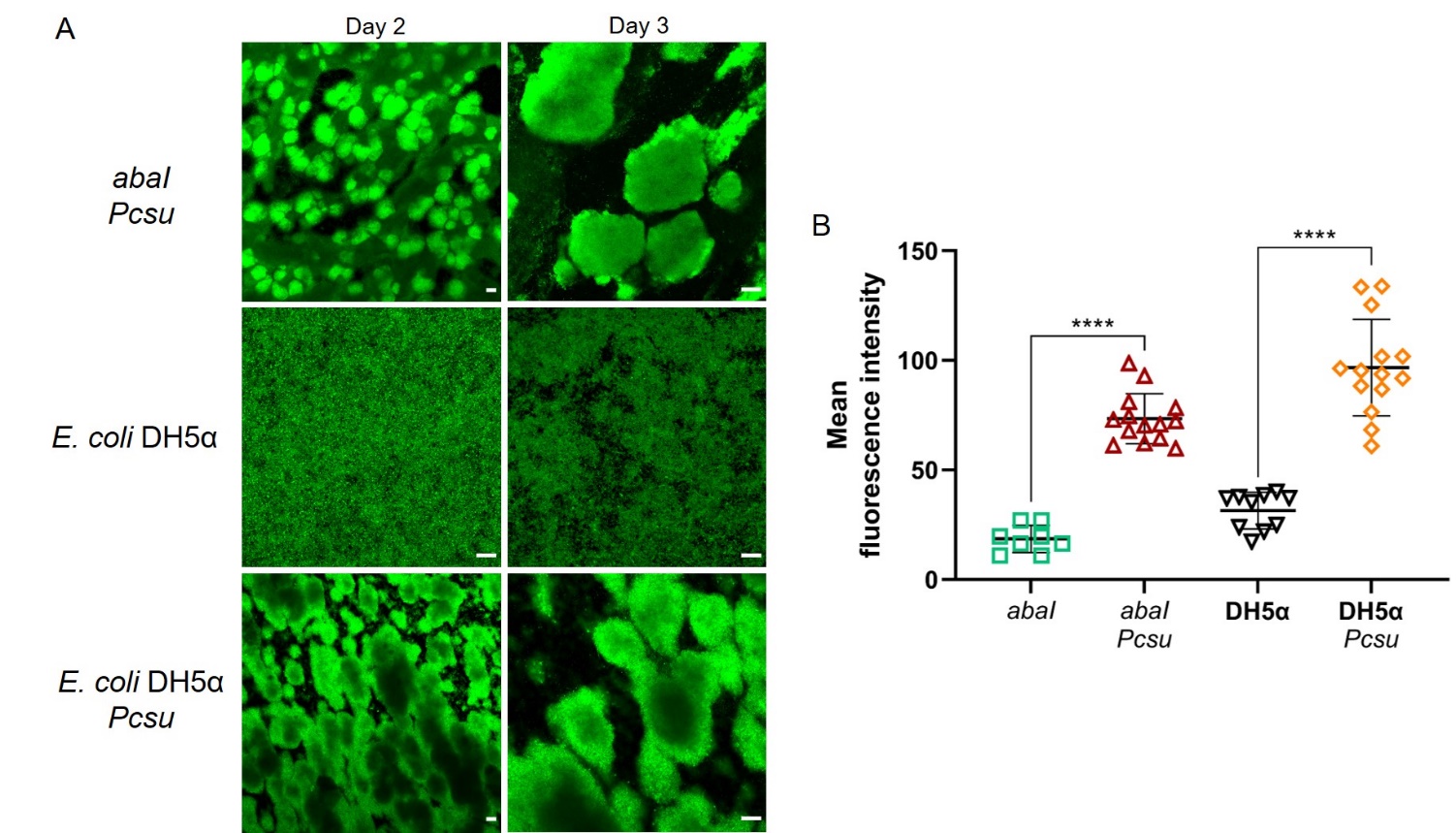
**

**Supplementary figure 4.** **A)** CLSM images of biofilm development of the *A. baumannii* *abaI* mutant strain constitutively expressing the *csu* operon (*Pcsu*) and *E. coli* DH5α -/+ csu after 2 and 3 days of incubation in the RBB and stained with Syto9. Scale bar: 50 µm. **B)** Quantification of mean fluorescence intensity of biofilm images from cultures of indicated strains after 3 days of incubation in the RBB and stained with Syto9. Data shown are mean ±SD. Statistical significance was determined with one-way Anova (* p<0.05; ** p<0.01; *** p<0.001; **** p<0.0001).
